# Flaxseed powder and magnesium hydroxide syrup on the intestinal function of patients with acute myocardial infarction in intensive care units 

**DOI:** 10.22088/cjim.15.2.234

**Published:** 2024

**Authors:** Sahar Amjadi Suraki, Masoumeh Bagheri-Nesami, Maryam Nabati, Mahmood Moosazadeh, Emran Habibi

**Affiliations:** 1 Student Research Committee, Mazandaran University of Medical Sciences, Sari, Iran; 2 Traditional and Complementary Medicine Research Center, Addiction Institute, Mazandaran University of Medical Sciences, Sari, Iran; 3 World Federation of Acupuncture-Moxibustion Societies (WFAS), Beijing, China; 4 Department of Cardiology, Cardiovascular Research Center, Mazandaran University of Medical Sciences, Sari, Iran; 5 Gastrointestinal Cancer Research Center, Non-communicable Diseases Institute, Mazandaran University of Medical Sciences, Sari, Iran; 6 Medicinal Plants Research center, Mazandaran University of Medical Sciences, Sari, Iran; 7 Department of Pharmacognosy and Biotechnology, School of Pharmacy, Mazandaran University of Medical Sciences, Sari, Iran

**Keywords:** Flaxseed, powder, Magnesium hydroxide syrup, Intestinal function, Acute myocardial infarction, Constipation.

## Abstract

**Background:**

Flaxseed powder seems to improve bowel movements in these patients. Therefore, this study compares the effects of flaxseed powder and magnesium hydroxide on bowel movements of acute myocardial infarction patients hospitalized in ICU.

**Methods:**

The population of the present parallel randomized controlled clinical trial included 70 acute myocardial infarction patients hospitalized in ICU who had no history of chronic constipation. The patients in the intervention group were given three sachets of flaxseed powder (each sachet was 3 g) twice a day for four days. The patients in the control group were given 20 cc of magnesium hydroxide syrup each morning. The Bristol scale was used to describe stool consistency.

**Results:**

The mean and standard deviation of the number of bowel movements within five days after intervention are 1.86 ± 1.08 and 1.6 ± 0.65 in the intervention and the control groups, respectively. The frequency of normal stool consistency of the first bowel movement is 94.3% for the intervention group and 85.7% for the control group, which shows no significant differences between the two groups in terms of stool consistency and bowel movement frequency (P=0.510). The bowel movements started on average after 35.2±97.97 hours in the flaxseed group and 24.771±2.677 hours in the magnesium hydroxide group (P=0.023).

**Conclusion:**

The results showed that flaxseed powder increases bowel movement frequency and improves the patients’ stool consistency, but the differences between the two groups are insignificant. Finally, the time to the first defecation was shorter in the magnesium hydroxide group.

Acute myocardial infarction (MI) is the most severe manifestation of coronary artery disease (1). Bowel disorder is very prevalent (15-82 percent) in cardiac patients hospitalized in ICU (2). Constipation is characterized by delayed or slower bowel movements without dysfunction of pelvic floor muscles (3). Some symptoms of this gastrointestinal disorder are hard and compact stools, the feeling of incomplete evacuation after defecation, the feeling of anorectal obstruction, and less than three times bowel movements per week for three months (4). Factors like absolute bed rest for a long time and embarrassment and difficulty of defecation in bed make MI patients prone to constipation. Some consequences of the Valsalva maneuver and difficult defecation in these patients are heart failure and the blood pressure rise (up to 70 mmHg), which may remain for an hour after defecation (5).

The stool pressure increases afterload and cardiac work, which is very dangerous in patients with a history of acute MI and may cause arrhythmias such as ventricular tachycardia and ventricular fibrillation. In other words, even if the patients have regular daily bowel movements, the difficulty of their defecation should be checked (5). Nowadays, laxatives are used to prevent and treat constipation. Laxatives based on their action mechanism are divided into five categories: bulk-forming laxatives (e.g., natural fibers like psyllium and bran), osmotic laxatives (e.g., magnesium hydroxide and magnesium sulfate), stimulant laxatives (e.g., bisacodyl), disaccharide laxatives (e.g., sorbitol and lactulose), and stool-softener laxatives (e.g., paraffin oil and docusate sodium). Each of these drugs has some side effects. Bulk-forming laxatives and disaccharides are flatulent. Stimulant and stool-softener laxatives can cause discomfort, cramps, and abdominal pain, and osmotic laxatives can cause water and electrolyte imbalance (6). Using laxatives like magnesium hydroxide by MI patients is not only not affordable but also leads to side effects (5). The use of magnesium hydroxide may cause hypermagnesemia, which its severe cases, especially in patients with kidney problems, can lead to death (7). In addition, hypermagnesemia may lead to changes in the electrocardiogram, such as prolonged PR, increased QRS duration, prolonged QT interval, and tall T-wave, which may lead to sinoatrial block and ventricular arrhythmias (8) and deteriorate the patients’ health. Therefore, MI patients should be cautious when using magnesium hydroxide syrup.

Complementary medicines such as acupuncture, massage therapy, and medicinal plants are currently used in addition to medical methods to treat constipation (6).

Some studies have investigated the effect of herbs such as rhubarb (9), psyllium, fig, and senna (6), prune (10), castor oil (11), and chicory (12) on constipation in different conditions. These herbs have side effects. Castor oil may cause water and electrolyte imbalances and acid-base imbalances in the body (11). Excessive (more than two months) use of chicory can change blood factors; especially, it can decrease hematocrit and MCV (13). ***Flaxseed***, a plant in the *Linaceae* family, has the scientific name *Linum usitatissimum* (14). It is an oily seed containing about 31.16% oil, 29.07% dietary fiber, and 20.86% protein (15). It has both soluble and insoluble fibers. Almost a quarter of the fibers are soluble, decreasing cholesterol and blood sugar. The rest of the fibers are insoluble, increasing bowel volume and facilitating defecation (16). Flaxseed mucilage is located in its outermost layer shell. When flaxseed gets wet, it forms a sticky layer (17). The mucilaginous part of flaxseed binds to water in the bowel, swells the stool, and thus increases the bowel volume. Linseed oil has a laxative effect by stimulating cholinergic and histaminergic receptors (18). In addition, flaxseed contains alpha-Linolenic acid (ALA), an essential fatty acid with potential biological effects like inhibiting platelet aggregation, reducing the risk of thrombosis, lowering blood pressure, reducing blood lipids, and preventing cardiac arrhythmias. Thus, it can prevent cardiovascular diseases and benefit MI patients (19). Research has shown that flaxseed powder outperforms lactulose in patients with chronic constipation (20). Another study investigating the preventive effect of flaxseed on constipation in healthy people found that flaxseed increases the stool’s volume and softness (21). The consumption of flaxseed powder and prunes in yogurt is shown to reduce constipation and abdominal pains caused by it (22). In addition, it is shown that baked flaxseed in type II diabetes patients with chronic constipation significantly improves (about 42%) their constipation (23). Another study showed that linseed oil does not relieve constipation better than olive and mineral oils in hemodialysis patients. Factors like the lack of influence of flaxseed oil on bowel movements(24), the risk of constipation in MI patients, and the side effects of magnesium hydroxide syrup (especially in kidney patients) prompted us to compare the effects of flaxseed powder versus magnesium hydroxide syrup on bowel movements in acute MI patients.

## Methods

This study is a parallel randomized controlled clinical trial. The university research ethics committee first approved the study with the code of IR.Mazums.Rec.1400.10347**. **Then, the study was registered in the Iranian Registry of Clinical Trials. The sample size was estimated based on the results of a similar study (20), in which the percentage of normal stool consistency before and after intervention in the flaxseed group was 21.7% and 86.7%, respectively. The sample size (54 patients) was determined using G-POWER and based on the above results and the formula of comparing two dependent ratios at the confidence level of 95% with the test power of 90%. The control and intervention groups included 27 patients, and after a 30% increase, the size of each group increased to 35 (i.e., a total of 70 patients).

Convenience sampling was performed with respect to the eligibility criteria on 70 heart attack patients admitted to ICU ward of Sari Fatemeh Zahra Hospital. The patients were hospitalized from September 2021 to May 2022. The inclusion criteria are the diagnosis of acute MI by a doctor, consent to participate in the study, less than 24 hours being passed since the patient admission to the hospital, no history of chronic constipation according to the Rome IV criteria, being over 18 years old, no history of allergies to medicinal plants, the ability of visual and verbal communication, no neurological and mental diseases, no thyroid, brain, liver, kidney diseases, none of the cancers, no digestive diseases such as Crohn's disease, irritable bowel syndrome, intestinal obstruction, no ulcers and bleeding in the gastrointestinal tract, no drug addiction, no pregnancy**, **no ejection fraction (EF) (less than 35%), not participating in other simultaneous studies, not using magnesium hydroxide syrup and any other laxatives within the last 24 hours. The exclusion criteria that stop the study are drug sensitivity during the research, deterioration or death of the patient, discharge or transfer of the patient to another hospital, using treatments for constipation other than flaxseed and magnesium hydroxide during the study, intolerance to the taste of flaxseed powder, and a score of 3 to 7 based on the Bristol scale.

The randomization permuted block procedure was used to randomly divide the patients into two groups. Each group included 35 patients, in total 11 blocks of six patients and 1 block of four patients, and accordingly, the samples were created.

 Seventy non-transparent and sealed envelopes were prepared, and letters A and B were assigned to flaxseed and magnesium hydroxide groups. The first envelope was opened with respect to the first eligible patient. Then, we filled out a questionnaire containing demographic and medical information of the patients, such as age, gender, height, weight, marital status, number of children, education level, occupation, place of residence, hospitalization history, history of previous diseases, initial pulse and blood pressure, ejection fraction (EF), the time elapsed since hospitalization, smoking status, dietary intake, history of using flaxseed, the use of any medicine or special food to facilitate bowel movements, mobility and the type and amount of antibiotics used in the last month. This study uses flaxseed powder sachets containing 3 g of flaxseed powder, produced by Zardband Pharmaceuticals, which has an ISO 9001 certificate, FAO organization certificate, and GMP certificate from the Iran Ministry of Health and Medical Education. The patients in the intervention group were given three sachets of flaxseed powder twice a day (22, 23) for four days (25, 26), and they were instructed to consume them at 9 am and 9 pm with 150 cc water (27) at night (20). The patients consumed six sachets of 3 g (18 g of flaxseed powder in total (21, 28) daily. The patients used the powder since the morning of the first day of their hospitalization for 96 hours after receiving the first dose (i.e., eight times in total). The preventive consumption of magnesium hydroxide syrup was started in the magnesium hydroxide syrup group after obtaining written informed consent on the first day of their hospitalization. The syrup used in this study is produced by Alborz Darou Company, which has ISO 9001 certificate and GMP standards certificate. The patients were instructed to take 20cc of the syrup dissolved in 300cc water (10) once a day at 9 pm for four days (29). The patients were given the medicine since the first night of their hospitalization for 96 hours (i.e., four times in total). If the drug prescribed for patients of both groups did not relieve their constipation five days after the intervention, the medication was changed. Clinical conditions of the patients and drugs to be consumed by them were determined 24 hours before the first intervention. In addition, their information 24, 48, 72, 96, and 120 hours after intervention were recorded.

The checklist included clinical conditions such as fluid intake, NPO duration, and the resting status of the patients. The daily bowel function of the patients 24 hours before the intervention and 24, 48, 72, 96, and 120 hours after the intervention were examined using a checklist based on the Rome VI criteria and the Bristol scale. The checklist included items such as straining during defecation, stool hardness, feeling of incomplete evacuation, feeling of anorectal obstruction, performing works by hand to facilitate defecation, and evaluating stool consistency. Various studies have confirmed the validity and reliability of this scale, and its reliability based on Cronbach's alpha, sensitivity, and specificity are 0.90, 78.6, and 82.9, respectively (30). Stool consistency based on the Bristol pictorial scale has the following seven categories:

1. Separate hard lumps (severe constipation),
2. Sausage-shaped but lumpy (mild constipation),
3. Sausage-shaped with cracks on the surface (normal defecation),
4. Sausage-shaped, soft, and smooth (normal defecation),
5. Soft blobs with a clear-cut edge (normal defecation),
6. Mushy stool (mild diarrhea), and
7. Watery without solid pieces (severe diarrhea) (31).

The researcher took pictures of ten patients' stools to determine the Bristol scale's inter-rater reliability. Then, the researcher and all the ward nurses evaluated the pictures, separately scored them, and calculated the correlation coefficient between them. Afterward, the intra-class correlation coefficient (ICC) was used to determine the coefficient of agreement between them (0.99), thus confirming their reliability. 

SPSS 25.0 was used to analyze the data. An independent t-test was used to compare the quantitative variables between the intervention and control groups. Chi-square and Fisher's exact tests were used to compare the grouped variables between the two groups. In addition, the Kaplan-Meier diagram and the log-rank test were used to compare the median duration of the intervention until the first defecation in both groups. Moreover, the Shapiro-Wilk test was used to examine the normality of quantitative variables.

**Figure 1:**
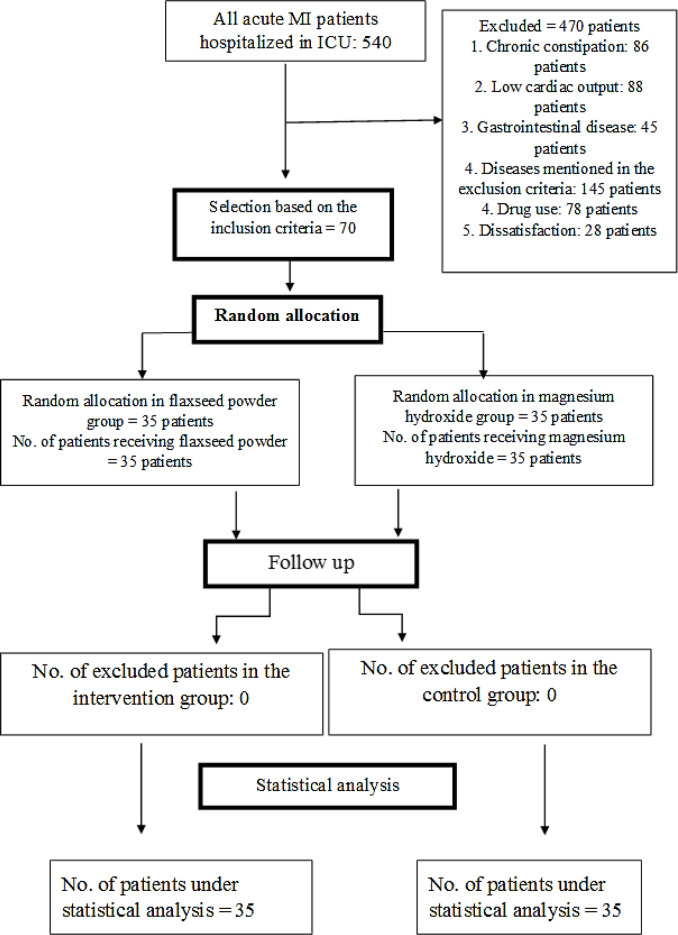
CONSORT flow diagram

## Results

Two groups were compared in terms of demographic and medical variables such as age, gender, body mass index, number of children, education level, occupation, place of residence, previous diseases, smoking, the time elapsed since hospitalization, dietary intake, using antibiotics during the last year, and no statistically significant difference was observed. 

The average ages in the intervention and the control groups were 63.57 ± 6.32 and 64.29 ± 9.37, respectively (P=0.710). The average body mass index in the intervention and the control groups were 27.48 ± 4.24 and 27.21 ± 4.88, respectively (P=0.810). In addition, the average duration of hospitalization until the first intervention in the intervention and the control groups were 13.74 ± 5.61 and 13.60 ± 5.42, respectively (P=0.914). Some patients had other diseases like blood pressure, increased blood lipids, heart ischemia, or diabetes (table 1).The bowel movement frequency of the patients was measured and recorded during the study and one day after the intervention (table 2). As shown in table 2, the F test showed no significant difference between the two groups regarding the number of bowel movements within five days after the intervention. In addition, table 3 compares the mean and standard deviation of the 24-hour bowel movements during the five days of the study. The mean and standard deviation of the bowel movement frequency for the patients in the intervention and control groups are 1.86±1.08 and 1.6±0.65, respectively. Thus, there is no significant difference between the groups (P=0.234). 

The Kaplan-Meier diagram (figure. 1) compares the times of the first bowel movements in both groups so that the medians of the control and intervention groups are 24±3.379 (second day) and 29±3.45 hours (second day) after the intervention, respectively. The results show that the average times to the first defecation in the control and intervention groups are 35.2±97.97 (confidence interval: 30.146-41.797 hours) and 24.771 ± 2.677 (confidence interval: 19.524-30.018) hours, respectively. The Log-rank test was used to compare the two groups, which showed that the time to the first defecation is significantly shorter in the control group (χ^2 ^= 5.144, df = 1, and P=0.023).

**Figure 2:**
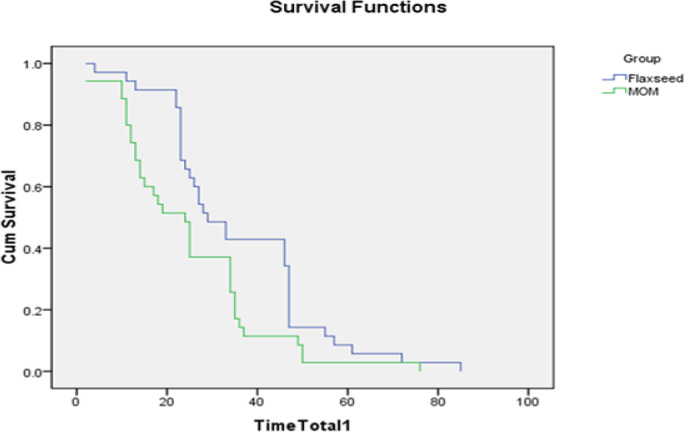
Persistent constipation probability with respect to the time of the first bowel movements for both groups

**Table 1:** Comparison of demographic and medical variables of patients with heart attack in intensive care in the two groups of intervention and control

**P- ** **value**	**Control**		**Intervention**		**group**	
	**Percent**	**Frequency**	**Percent**	**Frequency**	**Variable**	
*p=0/626	42/9%	15	37/1%	13	Male Female	**Gender**
	57/1%	20	62/9%	22		
*p=0/621	60%	21	65/7%	23	City Village	**Location**
	40%	14	34/3%	12		
*p=0/084	22/9%	8	5/7%	2	Yes No	**Smoker**
	77/1%	27	94/3%	33		
*p=1	85/7%	30	85/7%	30	Yes No	**History**
	14/3%	5	14/3%	5		
*p=0/077	77/1%	27	54/3%	19	Heart Diabetic	**Diet**
	22/9%	8	45/7%	16		
*p=0/628	37/1%	13	45/7%	16	Yes No	**Antibiotic**
	62/9%	22	54/3%	19		

* Chi-square, ** Fisher

**Table 2:** Comparison of the number of stool excretion patients with myocardial infarction five days after the onset of intervention in the two groups of intervention and control

**p** **value**	**MOM**		**Flaxeed**		**Groups** ** The time of review**	
	**Percent**	**Frequency**	**Percent**	**Frequency**		
**p=0/963	5/7%	2	5/7%	2	Everyday	24 hours before the start of the intervention
	25/7%	9	20%	7	Every second day	
	37/1 %	13	40%	14	Once every Two day	
	31/5%	11	34/3%	12	Once every three day	
**p=1	94/3%	33	91/4%	32	None	24 hours after intervention (day first)
	5/7%	2	5/7%	2	One	
	0%	0	2/9%	1	Two	
**p=0/901	37/1%	13	42/9%	15	None	48 hours after intervention (day 2)
	60%	21	54/2%	19	One	
	2/9%	1	2/9%	1	Two	
**p=0/631	57/1%	20	51/4%	18	None	72 hours after intervention (day 3)
	40%	14	48/6%	17	One	
	2/9%	1	0%	0	Two	
**p=1	77/1%	27	80%	28	None	96 hours after intervention (day 4)
	22/9%	8	20%	7	One	
**p=0/618	68/6%	24	60%	21	None	120 hours after intervention (day 5)
	31/4%	11	40%	14	One	

* Chi-square, ** Fisher

**Table 3:** Comparison of the average number of days of daily bowel patients with myocardial infarction within five days after the onset of intervention in the two groups of intervention and control

**Groups** **Defecation frequency**	**Flaxeed**	**MOM**
**Standard deviation ± average**	1/6 ± 0/65	1/86 ± 1/08
**p value**	*P=0/234	

* Chi-square, ** Fisher

Table 4 summarizes the stool consistency of patients with a heart attack for each day after intervention in both groups. In the intervention group, all the patients' bowel movements on the first and third days were normal (100%). On the second day, 20 patients had defecation, of which the stool consistency type of 19 (54.3%) patients was normal (type 5), and that of one (2.9%) patient was severe diarrhea (type 7). The stool consistency type in the intervention group was as follows on the fourth day: 6(17.1%) patients: type 5 (normal), and one (2.9%) patient: type 2 (i.e., mild constipation); and on the fifth day: 12 (34.3%) patients: type 5 (normal), and two (5.7%) patients: type 2 (i.e., mild constipation). In the control group, the bowel movement of all the patients with defecation on the first day was normal (100%). On the second day, 22 patients had defecation, of which the stool consistency type of 19 (54.3%) patients was normal (type 5), and that of 3(8.6%) patients had severe diarrhea (type 7). On the third day, 14 patients had defecation, of which the stool consistency type of 12 patients was type 5, that of 1 patient was type 7 (severe diarrhea), and that of another patient (2.9%) was type 2 (mild constipation). The stool consistency type in the control group was as follows on the fourth day: 4 (11.4%) patients: type 5 (normal), and three (8.6%) patients: type 2 (mild constipation); and on the fifth day: 6 (17.1%) patients: type 5 (normal), and five (14.3%) patients: type 2 (mild constipation). It should be noted that all the patients had normal stool consistency before hospitalization.

Table 5 presents the frequency of the first stool consistency for both groups within five days after the intervention. This table shows that one patient in the intervention group and two patients in the control group had constipation. In addition, the stool consistency type of 33 patients in the intervention group and 30 patients in the control group was normal (type 5). Moreover, one patient in the intervention group and three patients in the control group had severe diarrhea (type 7). The seven types of data were merged into three types for statistical analysis: types 1 and 2 as constipation, types 3, 4, and 5 as normal, and types 7 and 8 as diarrhea. However, there was no significant difference between the two groups in the merged groups (P= 0.510). 

**Table 4:** Comparison of the Frequency of Stool Form of Patients with Myocardial infarction by Day, after the onset of intervention in the two groups of intervention and control

**P-value**	**MOM**		**Flaxeed**		**Groups** **The time of review**	
	**Percent**	**Frequency**	**Percent**	**Frequency**		
**p=1	94/3%	33	91/3%	32	No Defecation	24 hours after intervention (day 1)
	0%	0	0%	0	Type 2 (constipation)	
	5/7%	2	8/6%	3	Type 5 (normal)	
	0%	0	0%	0	Type 7 (Diarrhea)	
**p=0/694	37/1%	13	42/9%	15	No Defecation	48 hours after intervention (day 2)
	0%	0	0%	0	Type 2 (constipation)	
	54/3%	19	54/2%	19	Type 5 (normal)	
	8/6%	3	2/9%	1	Type 7 (Diarrhea)	
**p=0/726	57/1%	20	51/4%	18	No Defecation	72 hours after intervention (day 3)
	2/9%	1	2/9%	1	Type 2 (constipation)	
	34/2	12	45/7%	16	Type 5 (normal)	
	2/9%	1	0%	0	Type 7 (Diarrhea)	
**p=0/598	80%	28	80%	28	No Defecation	96 hours after intervention (day 4)
	8/6%	3	2/9%	1	Type 2 (constipation)	
	11/4%	4	17/1%	6	Type 5 (normal)	
**p=0/191	68/6%	24	60%	21	No Defecation	120 hours after intervention (day 5)
	14/3%	5	5/7%	2	Type 2 (constipation)	
	17/1%	6	34/2%	12	Type 5 (normal)	

**Table 5:** Comparison of the Stool Form First Patients with Myocardial infarction within five days after the intervention begins in the two groups of intervention and control

**Groups** **Stool Form**	**Flaxeed**		**MOM**	
	**Frequency**	**Percent**	**Frequency**	**Percent**
**Type 1 and 2 (constipation)**	1	2/9%	2	5/7%
**Type 3, 4 and 5 (normal)**	33	94/3%	30	85/7%
**Type 6 and 7 (diarrhea)**	1	2/9%	3	8/6%
**P value**	**p=0/510			

* Chi-square, ** Fisher

## Discussion

This study compares the effects of flaxseed powder and magnesium hydroxide syrup on bowel movements of acute MI patients hospitalized in ICU wards. The results show that flaxseed powder can increase the frequency of bowel movements and stool consistency in these patients. However, there was no significant difference between the two groups. The results showed that the time from the start of the intervention to the first bowel movements was shorter in the magnesium hydroxide group. To the best of our knowledge, there is no study comparing magnesium hydroxide and flaxseed, but there are studies on the effect of magnesium hydroxide on bowel movements. For example, a study has investigated the effects of prune and magnesium hydroxide on constipation among 24 stroke patients hospitalized in ICU who were constipated. The patients in the intervention group were given 50 g of prune, and those in the control group were given 30cc of magnesium hydroxide syrup using a gavage tube twice a day for two days. They recorded the bowel movement frequency and stool consistency of the patients in both groups and found similar results. In addition, they found that the time to the first bowel movements for both groups is equal. Moreover, the stool consistency type in the syrup group compared to that in the prune group was further diarrhea (10), which is consistent with our results. The syrup increased the average frequency of bowel movements, but also increased diarrhea. 

Another study compared the effects of Golqand (a combination of Rosa, damascena Herrm, and honey) and magnesium hydroxide syrup on chronic constipation. This clinical trial assigned 56 patients with chronic constipation to two groups randomly. The first group took 20 ml of magnesium hydroxide in the morning and 20ml at night before bed. The second group dissolved 10 g of Golqand in lukewarm water and swallowed it before lunch and another 10 g before dinner. Then, they did not use any medication in the second two weeks. The patients were examined before the study and two weeks and four weeks after the start of the study. The average bowel movement frequency in the first group was higher than in the Golqand group in the first two weeks. However, there was no significant difference between the two groups in the third week, i.e., when the treatment was stopped. However, the frequency of bowel movements in the Golqand group was higher than that in the magnesium hydroxide group in the fourth week. As a result, although magnesium hydroxide is more effective than Golqand in the first weeks, the effects of Golqand are more durable (32). 

The present study might also achieve similar results by increasing the follow-up period after the intervention. Although they examine the patients with chronic constipation for a more extended period, the present study examines patients with acute constipation for a shorter period. 

There are studies on the laxative properties of flaxseed to prevent and treat chronic constipation. A study investigated the effect of flaxseed powder on stool volume and blood sugar levels in healthy volunteers. They instructed the participants to consume small packets containing 5 g of flaxseed powder three times a day for two weeks (in total, 15 g of flaxseed powder per day). The study period was two weeks, and the patients were given stool collection containers to collect their stools. Then, the weight of the collected stools during the basic and treatment periods was examined. The results showed that flaxseed powder could significantly increase bowel movement frequency and stool consistency (33), which is consistent with our results. They have examined chronic constipation, but the current study examines acute constipation. 

Another study investigated the effect of olive oil and flaxseed oil on chronic constipation in hemodialysis patients. They divided 68 hemodialysis patients into three groups: the first group (16 patients) consumed olive oil, the second group (17 patients) consumed linseed oil, and the third group (17 patients) consumed mineral oil. Each patient consumed four cc of the oil daily for four weeks. Then, they examined symptoms of constipation concerning the Rome III criteria and Bristol scale. As a result, constipation scores decreased in all groups, indicating that daily use of all three oils effectively treats constipation in hemodialysis patients. However, the flaxseed oil was less effective than the olive and mineral oils (24). 

This study shows that the fiber existing in flaxseed probably has a significant effect on its laxative properties. Another study investigated the effect of flaxseed on chronic constipation, blood sugar, blood lipid, and weight of patients with type 2 diabetes. The participants in the intervention group ate four cookies containing 2.5 g of flaxseeds (10 g daily in total) with orange flavor for 12 weeks. The Rome III criteria indicated that the average constipation symptoms in the intervention group compared to the control group were reduced significantly (23), which is consistent with our results. Another study investigated the effect of flaxseed powder on chronic constipation and quality of life in a Chinese population. The first group was given 50 g of flaxseed powder daily, and the second group was given 15 ml of lactulose syrup each day. This study lasted four weeks. The results showed that flaxseed powder is more effective than lactulose syrup in increasing the frequency of bowel movements (20). This study examines hemodialysis patients with chronic constipation, while the current research examines MI patients with acute constipation. This study showed that flaxseed powder effectively improves bowel movements in MI patients.

In this study, bowel movements increased in both groups after the intervention. However, the chi-square test did not show a significant difference between two groups. The number of patients with normal stool consistency in the first bowel movements in the flaxseed group was more than that in the magnesium hydroxide group. In addition, the number of patients with diarrhea (i.e., types 6 and 7) was more in the magnesium hydroxide syrup group. The number of patients with the stool consistency of types 1 and 2 (i.e., constipation) was more in the magnesium hydroxide syrup group; however, no statistically significant difference was observed between the two groups. The results showed that the time to the first bowel movements after the intervention was shorter in the control group. 

There are many effective variables that are beyond the researcher's control. For example, this research was conducted during the corona pandemic and the stress of this issue was added to the stress caused by illness and hospitalization. Since stress affects intestinal function, this variable could not be controlled by the researcher. To the best of our knowledge, the current study is the first to examine the effects of flaxseed powder on bowel movement frequency in MI patients hospitalized in ICU. Therefore, we suggest conducting studies with a more significant number of patients in this context.
